# Post-infectious ankylosis of the cervical spine in an army veteran: a case report

**DOI:** 10.1186/s12998-018-0211-1

**Published:** 2018-10-30

**Authors:** Zachary A. Cupler, Michael T. Anderson, Eric T. Stefanowicz, Chad D. Warshel

**Affiliations:** 1 Physical Medicine & Rehabilitative Services, VA Butler Healthcare, 353 North Duffy Road, Butler, PA 16001 USA; 2 Department of Chiropractic Clinical Sciences, New York Chiropractic College, Seneca Falls, USA

**Keywords:** Ankylosis, Cervical, Veteran, Military personnel, Osteomyelitis, Spondylodiscitis

## Abstract

**Background:**

Vertebral osteomyelitis is a rare, life-threatening condition. Successful management is dependent on prompt diagnosis and management with intravenous antibiotic therapy or surgery in addition to antibiotics. Reoccurrence is minimal after 1 year. However, very little is reported in the conservative spine literature regarding the long-term follow-up and the changes to the spine following management of the spinal infection. We report the dramatic radiologic findings of the long-term sequela of a cervical spine infection following a gunshot wound from 1969. Most impressive to the spine specialist is this patient’s ability to return to work despite significant alterations to spinal biomechanics.

**Case presentation:**

A 69 year-old caucasian male presented to the chiropractic clinic at a Veterans Affairs Medical Center with complaint of chronic left shoulder pain secondary to an associated full thickness tear of the left infraspinatus. An associated regional assessment of the cervical spine ensued. Radiological imaging on file revealed ankylosis C2/C3 to C7/T1. The patient reported a history of multiple fragment wounds in 1969 to the left anterior neck and shoulder 45 years earlier. Osteomyelitis of the cervical spine resulted from the wounds.

**Conclusion:**

Potential sequela of osteomyelitis is ankylosis of affected joints. In this particular case, imaging provides evidence of regional ankylosis of the cervical spine. Considering the patient did not complain of cervical pain or related symptoms apart from lack of cervical range of motion, and his Neck Disability Index score was 2 out of 50 (4%), no intervention was provided to the cervical spine. The patient reported he self-managed well, worked full-time as a postal worker after he was discharged due to the injury to his neck, and planned to retire in less than one month at age 70. The patient demonstrates successful return to work with pending retirement at age 70 following spondylodiscitis and subsequent ankylosis of the cervical region.

## Background

Spondylodiscitis, or vertebral osteomyelitis (VO), is specifically an infection of the spine, primarily the intervertebral disc and adjacent vertebral bodies. Prompt diagnosis is necessary for improved outcomes whether managed with antibiotics, surgery, or a combination of both. The majority of spondylodiscitis cases are treated conservatively with 10–20% managed surgically [1]. Surgical management is indicated in cases when there is a disease reoccurrence despite appropriate medical management with antibiotic therapy, neurological deficit, intractable pain, epidural abscess, as well as progressive vertebral body destruction and/or spinal deformity [2, 3].

The incidence of VO has risen in recent years with increased risk correlated with co-morbidities and serious medical illness [3]. Previous reports have indicated cervical spine VO accounts for 3 to 11% of VO cases [2]. Management of VO resulting from gunshot wounds to the neck have previously been described in the literature [4–7].

However, long-term follow-up reports with imaging secondary to conservatively managed VO is a rarity. Garcia and Grantham reported that the average time to return to work was 12 months and spontaneous interbody fusion was the rule rather than the exception [8]. Neck pain is ranked as the fourth leading cause of years lived with disability [9]. Compared to a reference population, patients with infectious spondylodiscitis have demonstrated a reduced ability to work after the infection [10]. We present the imaging findings of an individual who suffered from VO 45 years earlier and continued to work full-time.

## Case presentation

A 69 year-old Caucasian male Army veteran was referred to a chiropractor at a Veterans Affairs Medical Center. He presented with left shoulder pain in the setting of a full thickness supraspinatus tear. A thorough history of his neck was gathered as it related to the left shoulder pain. His cervical spine was “stiff” most mornings, which abated with movement and activity. The patient had limited cervical range of motion in all planes and noted episodic neck pain secondary to injuries he sustained from multiple fragment wounds to left side of his neck in 1969.

A review of the final field hospital narrative at the time of initial trauma revealed the multiple frag wounds to the neck, shoulder and scrotum. These injuries subsequently resulted in a trachea-esophageal cutaneous fistula with left cervico-mediastinal abscess and cervical VO. During hospitalization, cervical plain films were reported on which described “prominent demineralization of the bones of the cervical spine with decrease in disk spaces C2 through C6. More demineralization anteriorly with apparent destruction of the anterior aspect of vertebral bodies with resultant reversal of normal lordotic curve. Neural foramina appear intact.” There was no mention of zygapophyseal joint fusion in the original radiographic reports following injury. He was hospitalized for 17 months following the gunshot wound with multiple surgeries to debride the region and reconstruct the left cervical musculature as well as antibiotic therapy. Cervical plain films at time of discharge described “bony healing and fusion of the mid-cervical spine with fusion of 2^nd^ through 5^th^ cervical bodes and calcification of the anterior ligament, C5-C6, C6-C7”. Again there was no remark of zygapophyseal joint fusion after treatment for the initial injuries and subsequent infection.

In office, he endorsed occasional axial neck pain and occipital headache that occurred 1 time per week. Neck Disability Index (NDI) score was 2 out of 50 (4%) [11]. Pertinent details from the review of systems revealed type 2 diabetes mellitus, carpal tunnel syndrome, hypothyroidism, and irritable bowel syndrome. His medications included tramadol and butalbital, both taken as needed. He used a Thera Cane for self-management of myofascial pain. Despite this history, the patient worked full time from his 20s and was planning to retire at the age of 70.

Physical exam findings revealed a man of anticipated age who was oriented to person, place and time. He weighed 82 kg (181.2 lbs) and measured 177 cm (70 in.). Neurologic examination of the upper and lower extremity dermatomes revealed no deficits. Romberg test was negative and failed to elicit body sway or sense of loss of balance. Biceps, triceps and brachioradialis deep tendon reflexes were 2+ when elicited bilaterally. The plantar response was down going and symmetric. Finger to nose movements were without dysmetria or tremor. Cervical rotation was found to be severely limited in both directions. Global restriction of the cervical spine was noted when assessing joint play, with no isolated segmental motion.

**Fig. 1:**
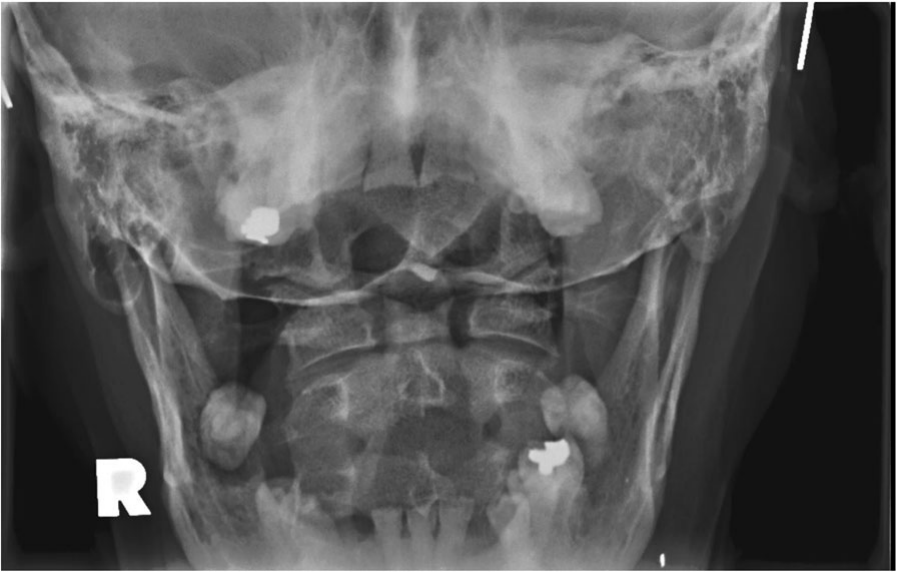
AP Open Mouth Plain Films

**Fig. 2:**
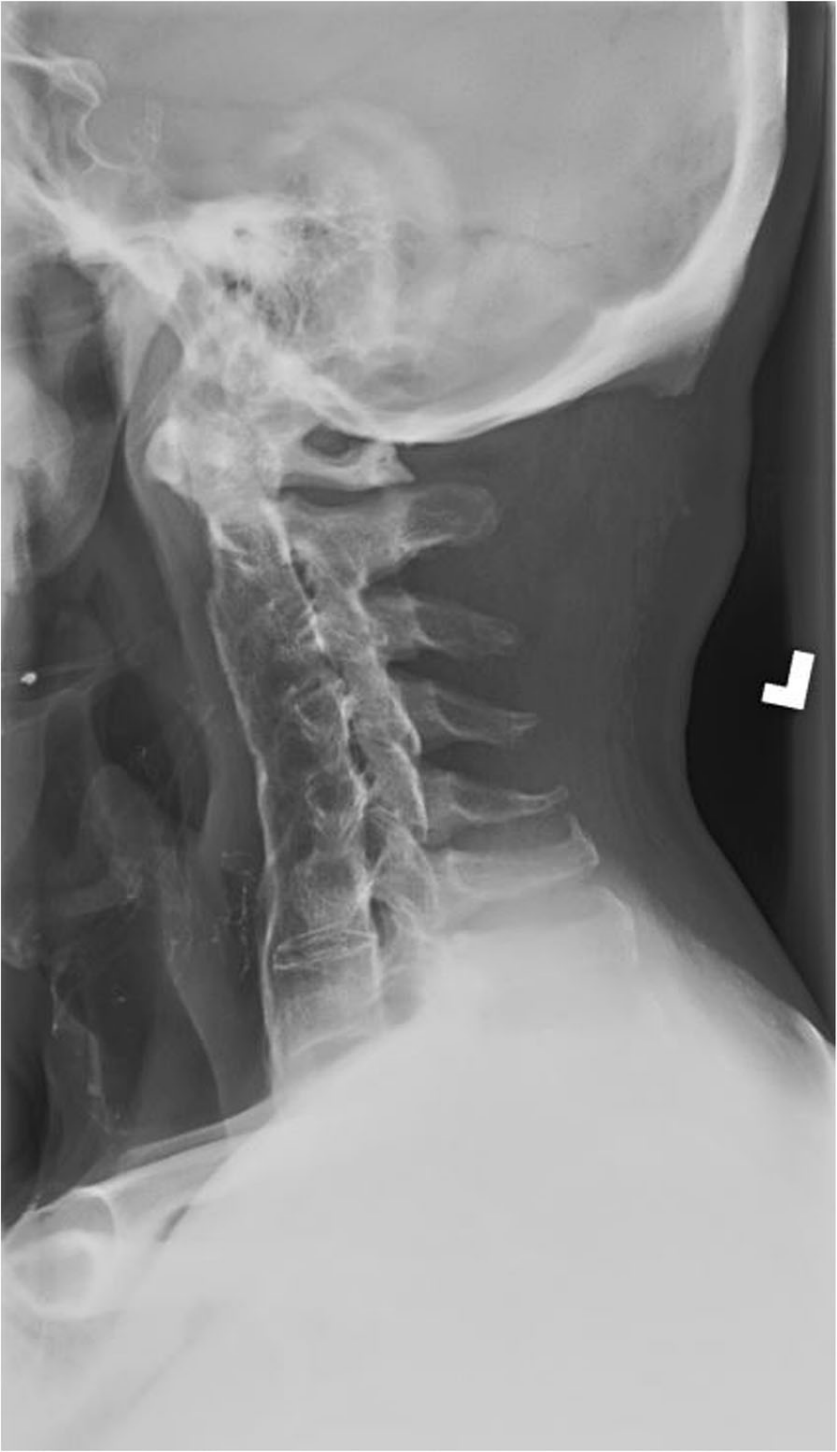
Lateral Cervical Plain Films

**Fig. 3:**
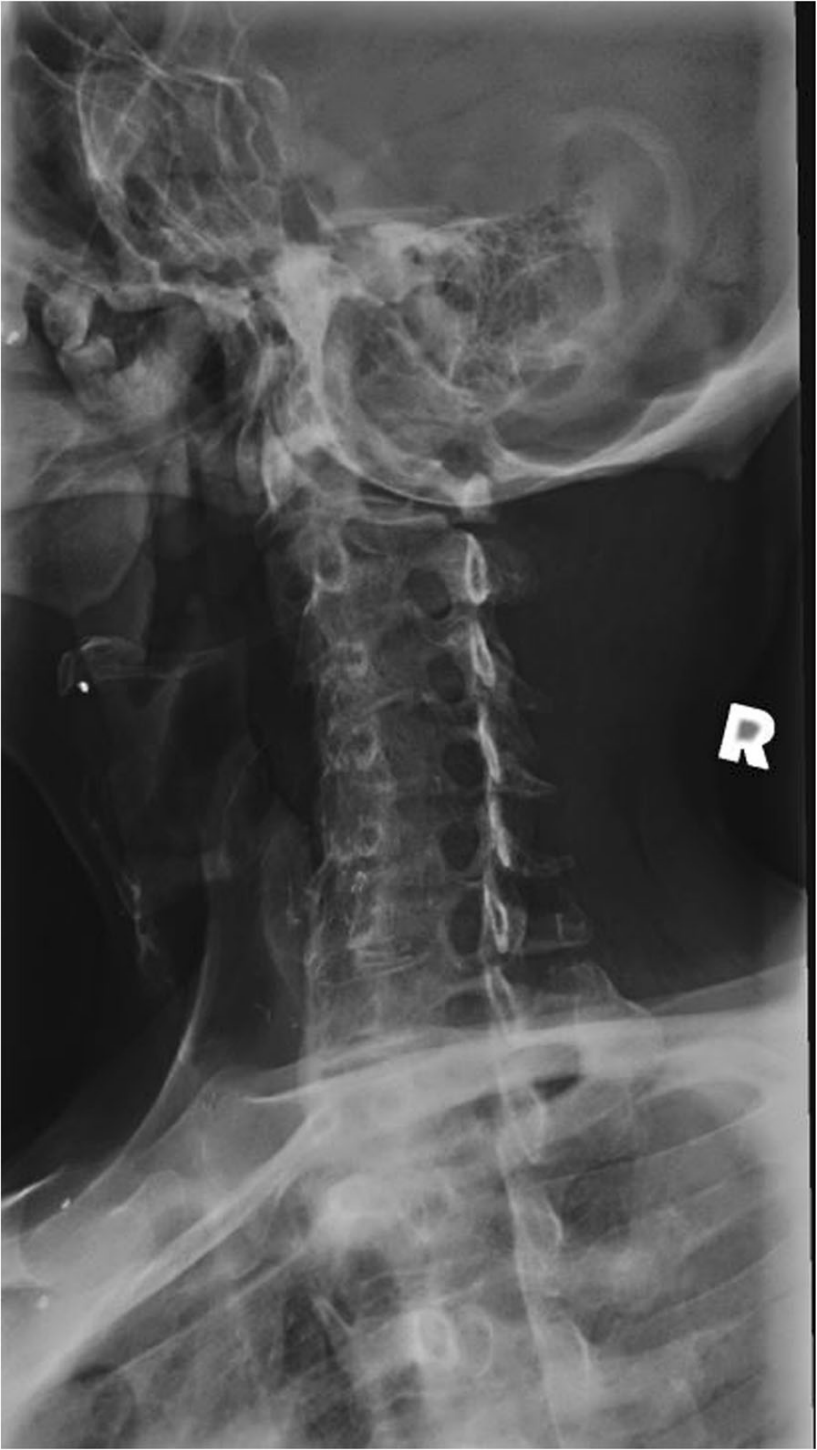
Right Oblique Plain Films, patent intervertebral foramina visualized

**Fig. 4:**
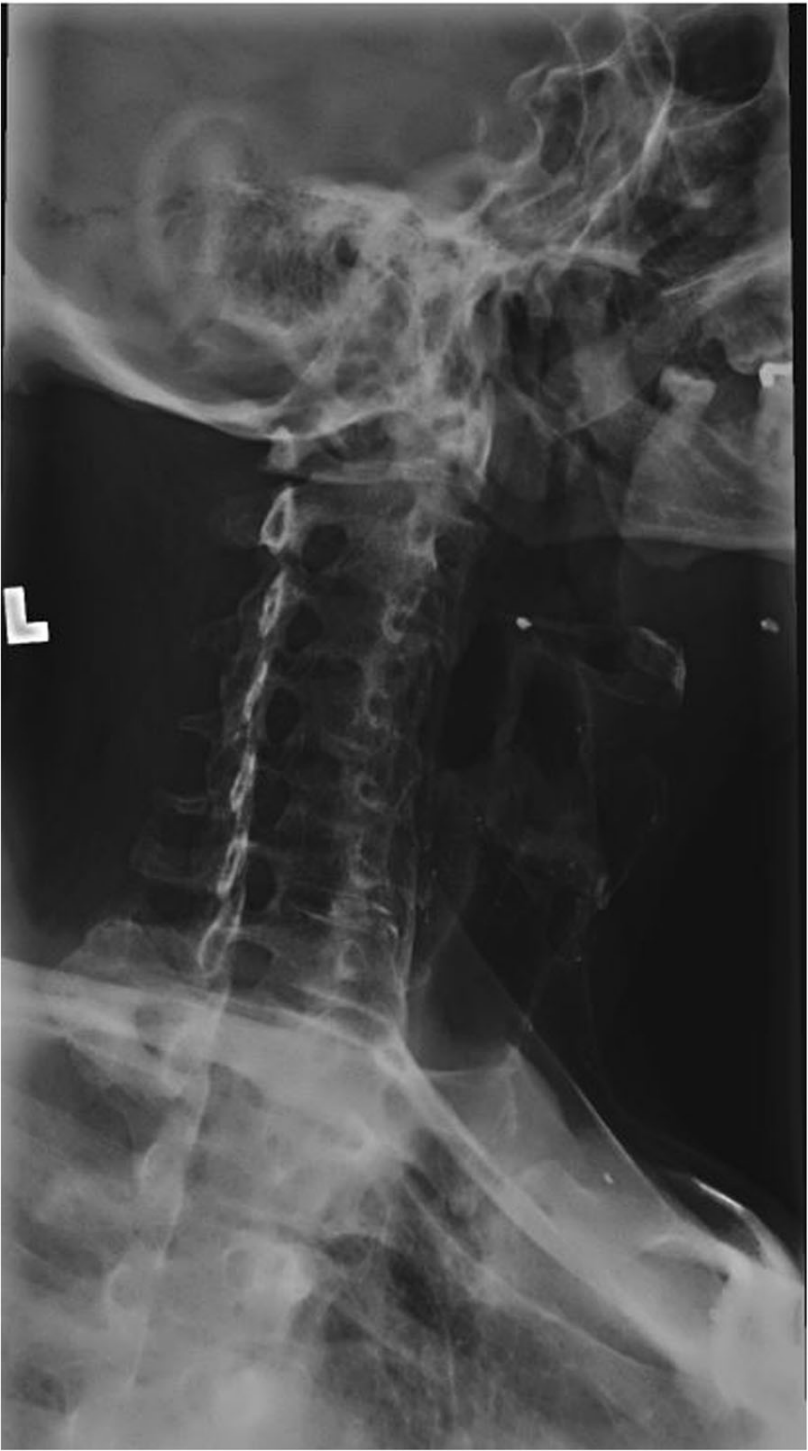
Left Oblique Plain Films, patent intervertebral foramina visualized

**Fig. 5:**
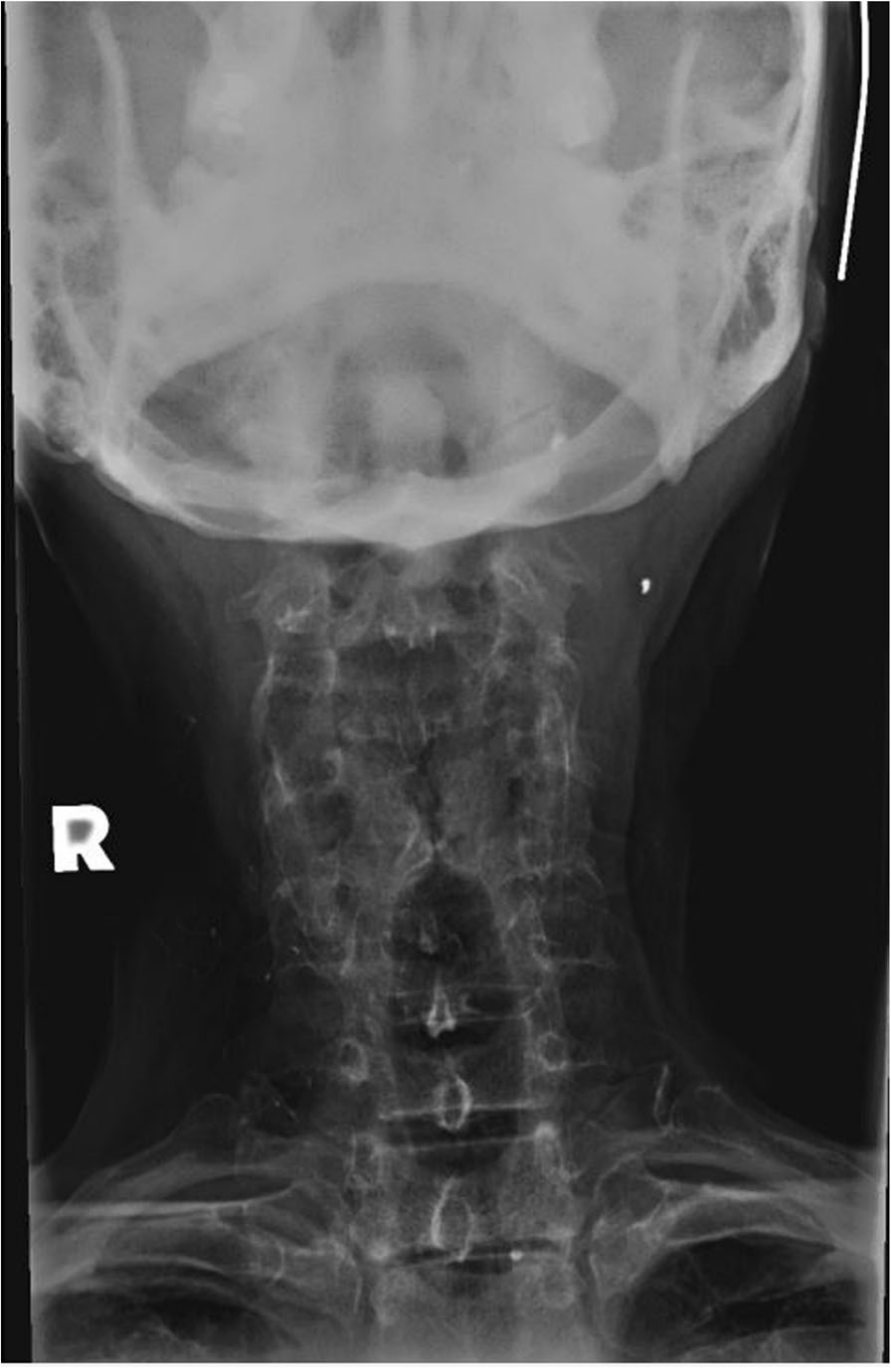
AP Cervical Plain Films

Radiographs of the cervical spine on file demonstrate osseous ankylosis from C2/C3 to C7/T1 with obliteration of the intervertebral discs and accompanying endplates from C2/C3 to C5/C6. (Figs. 1, 2, 3, 4, 5) The C6/C7 and C7/T1 intervertebral discs and accompanying endplates are visualized, though the levels are ankylosed. The facet joints from C2-C6 are ankylosed as well. There is a mild kyphotic alignment of the cervical spine. Metallic fragments are seen in the soft tissues of the neck and upper thorax consistent with the stated history. Provided the history of osteomyelitis secondary to the treatment of the wound and multiple surgeries, the most likely diagnosis is post-infectious ankylosis from C2-T1. The initial field narrative did describe spondylodiscitis with observation of fusion of multiple segments of the cervical spine upon discharge 17 months later. No clear infection of the facet joint was described at the time. Traditional spondylodiscitis is observed to be contained to the anterior column of the spine, primarily the vertebral endplate and adjacent intervertebral disc. However, it has been reported, when infection involves a vertebral body, that it may extend into the pedicles and articular processes which may result in adjacent septic facet arthritis [12].

No manual care to the cervical spine occurred following the evaluation as the patient had no complaint in addition to the NDI score of 4%. There was no indication for additional imaging or further work-up regarding the cervical spine. The patient reported complete functional independence in all activities of daily living with reasonable expectation of associated stiffness and limited cervical range of motion. Following the evaluation, the extent of care included education to the patient. Further work-up for the left shoulder and referral to physical therapy resolved left shoulder pain.

## Discussion

In infectious spondylodiscitis, with early treatment and management, preservation of disc height can be achieved, in addition to healing with ankylosis [8, 13, 14]. If diagnosis is delayed, ankylosis remains a late complication and sequela that may be encountered [15–17]. Non-operative management results in spontaneous inter-body fusion in 36–100% of cases [18–21]. Partial or total interbody fusion of involved vertebrae has been observed most commonly in the cervical spine (100%) followed by the thoracic (75%) and then lumbar spine (23%) [7].

**Fig. 6:**
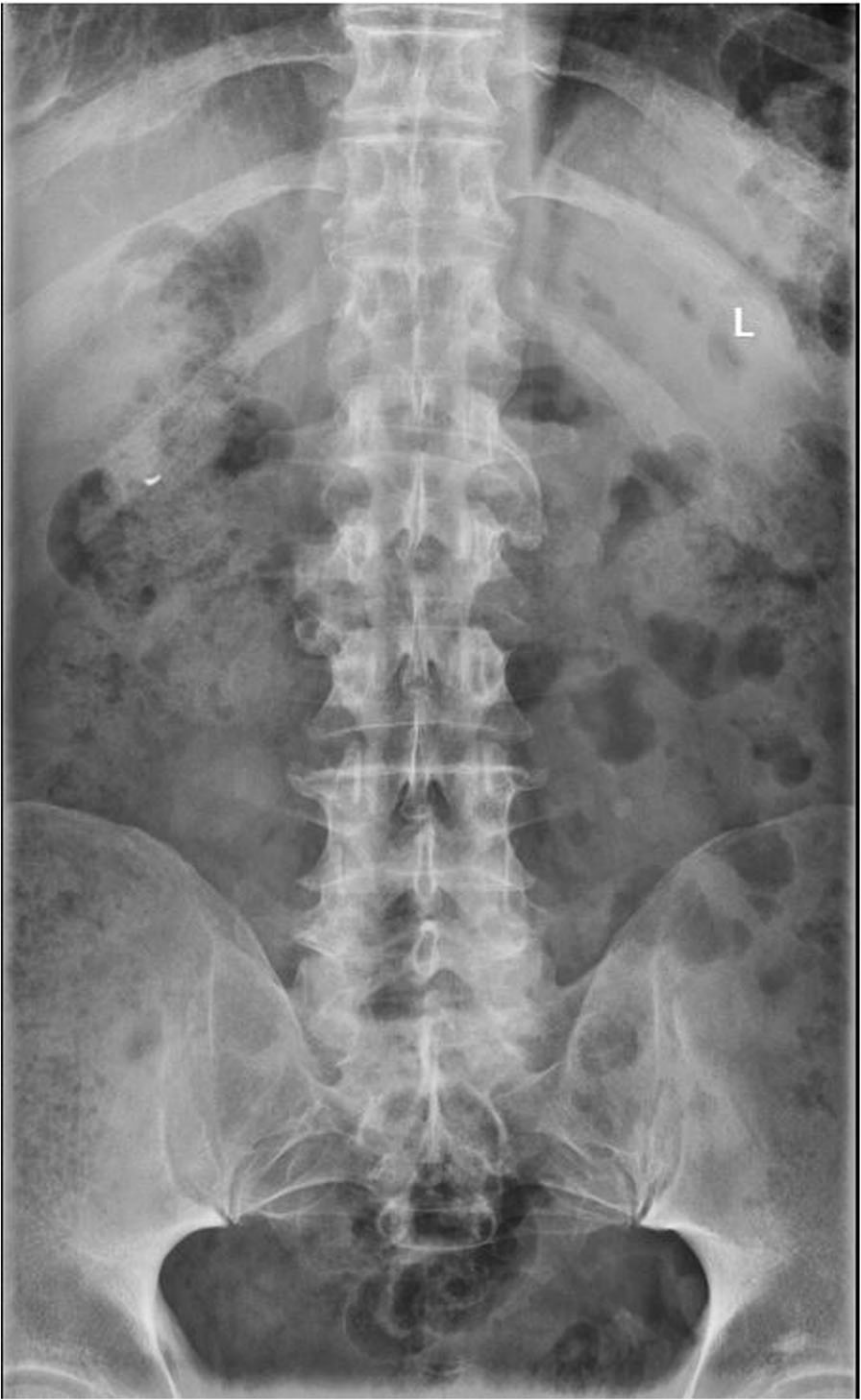
AP Lumbar Plain Films; preserved sacroiliac joint lines

Although the patient has a history of irritable bowel disease, this is not distinguished as an inflammatory bowel condition such as ulcerative colitis or Crohn’s disease which are associated with seronegative spondyloarthropathies. It is unlikely that this represents a seronegative spondyloarthropathy due to the obliteration of intervertebral discs and adjacent vertebral endplates as well as sparing of the remainder of the spine and sacroiliac joints. (Fig. 6) Diffuse idiopathic skeletal hyperostosis is not felt to be an appropriate differential as there is obliteration of intervertebral discs and endplates and there is no flowing ossification of the anterior longitudinal ligament, regardless of a history of diabetes mellitus. A differential consideration must include juvenile idiopathic arthritis, primarily a conglomerate of rheumatoid factor negative conditions [22], due to predilection for involving the cervical spine intervertebral discs and facet joints. This is less likely due to the degree of disc obliteration and the absence of hypoplastic vertebral bodies and absence of serologic markers.

### Limitations

This report describes a single case with relationship to an injury that occurred greater than 40 years ago. All abnormalities of the cervical spine cannot be assumed to be related to the initial injury leading to infection, as the natural progressive degenerative changes are not controlled for. Causation regarding the facet fusion is limited without original images to review. There is evidence of regional segmental body ankylosis on radiographs described in the final narrative reports from initial discharge records following infection without mention of the state of the cervical and thoracic region’s zygapophyseal joints. In this case, it is plausible and highly likely that hematogenous spread occurred from the abscess to the intervertebral discs and facet joints [12].

Only pre-existing historic clinical records, imaging and laboratory values were available for case review. While academically interesting, there was no clinical indication for additional imaging or lab work. There is no clinical history or evidence of prior rheumatological work-up to definitively exclude inflammatory arthropathies. No magnetic resonance or computed tomography images were available to review to further assess the integrity of the spinal cord, though neurological examination was unremarkable.

### Consent

Written informed consent to publish has been obtained from the individual for publication of their individual details and accompanying images in this manuscript.

## Conclusion

Spontaneous interbody ankylosis of the effected segments following spondylodiscitis is an expected progression of the condition. The spine practitioner should be aware of this response to spondylodiscitis when the patient is managed non-surgically. Additionally, the spine practitioner can positively influence patients to return to work with an encouraging prognosis despite such a diagnosis. This case is exceptionally unique because, unexpectedly, the facet joints of the effected segments are observed to be ankylosed as well without observed spinal cord involvement. While the state of the zygapophyseal joints at the time of initial treatment is not known, it is plausible that hematogenous spread occurred from the abscess to the intervertebral discs and facet joints.

## Abbreviations

NDI: Neck disability index
VO: Vertebral osteomyelitis
